# An inter-laboratory study characterizes the impact of bioinformatic approaches on genome-based cluster detection for foodborne bacterial pathogens

**DOI:** 10.3389/fmicb.2025.1629731

**Published:** 2025-11-03

**Authors:** Leonie F. Forth, Burkhard Malorny, Markus Bönn, Erik Brinks, Grégoire Denay, Carlus Deneke, Hosny El-Adawy, Jennie Fischer, Jannika Fuchs, Ekkehard Hiller, Nancy Bretschneider, Sylvia Kleta, Stefanie Lüth, Tilman Schultze, Henning Petersen, Michaela Projahn, Christian Schäfers, Kerstin Stingl, Andreas J. Stroehlein, Laura Uelze, Kathrin Szabo, Anne Wöhlke, Jörg Linde

**Affiliations:** ^1^Department of Biological Safety, German Federal Institute for Risk Assessment, Berlin, Germany; ^2^State Office for Consumer Protection of Sachsen-Anhalt, Halle (Saale), Germany; ^3^Institute of Microbiology and Biotechnology, Max Rubner-Institut, Kiel, Germany; ^4^Chemical and Veterinary Analytical Institute Rhein-Ruhr-Wupper (CVUA-RRW), Krefeld, Germany; ^5^Institute of Bacterial Infections and Zoonoses, Friedrich-Loeffler-Institut, Jena, Germany; ^6^Chemical and Veterinary Analysis Agency Karlsruhe, Karlsruhe, Germany; ^7^Chemical and Veterinary Analysis Agency Stuttgart, Fellbach, Germany; ^8^Bavarian Health and Food Safety Authority, Oberschleißheim, Germany; ^9^Department of Veterinary Medicine, Hessian State Laboratory, Giessen, Germany; ^10^Chemical and Veterinary Analytical Institute Ostwestfalen-Lippe, Detmold, Germany; ^11^Hamburg Public Laboratories for Food Safety, Health Protection and Environmental Assessment, Institute for Hygiene and Environment, Hamburg, Germany; ^12^Sequencing and Genotyping Service Unit, Max Planck Institute of Molecular Cell Biology and Genetics (MPI-CBG), Dresden, Germany; ^13^Department Official Collection of Methods of Analysis (ASU), Federal Office of Consumer Protection and Food Safety, Berlin, Germany; ^14^Food and Veterinary Institute Braunschweig/Hannover, Lower Saxony State Office for Consumer Protection and Food Safety, Braunschweig, Germany

**Keywords:** inter-laboratory study, whole-genome sequencing, public health, food safety, bioinformatic tools, cluster detection, foodborne pathogens

## Abstract

Accurate assignment of whole-genome sequences to clusters in foodborne outbreak investigations remains challenging. Variability in bioinformatics tools and quality metrics significantly impacts clustering outcomes. This study assessed inter-laboratory variance in cluster identification by providing four datasets of 50 raw Illumina paired-end sequences covering Shiga toxin-producing *Escherichia coli, Listeria monocytogenes, Salmonella enterica*, and *Campylobacter jejuni*. Following general rules of a specified guideline, participants applied in-house protocols for read quality assessment, 7-gene MLST, cgMLST, and SNP calling, then assigned samples to predefined focus clusters based on allele distance (AD) and mutations. Results revealed that differences in the interpretation of raw sequence and genome assembly quality influenced sample inclusion and finally cluster composition. Here, intra-species contamination was the most significant factor driving variability in decisions on whether to include or exclude samples. With one exception, 7-gene Multilocus-Sequence Typing (MLST) yielded consistent sequence types using different bioinformatics tools. The largest influence on cgMLST-defined clusters was the inclusion or exclusion of samples. Regarding bioinformatics, cgMLST was mainly reproducible. For *S. enterica*, discrepancies due to different software (Ridom SeqSphere+ vs. ChewieSnake) were larger than discrepancies due to different schemas. For other species, different schemas introduced larger discrepancies than different software. Most notably, *C. jejuni* cluster assignment was strongly affected by cgMLST schemas differing by a factor of two in the number of loci. SNP calling using Snippy produced concordant results across participants, except for *C. jejuni* when recombination filtering was used. This study highlights the impact caused by different interpretations of quality values when assessing clusters. Low-resolution cgMLST schemas were unsuitable for *Campylobacter jejuni*, and clustering near cut-off values was sensitive to bioinformatics tool selection. Standardized protocols are essential for reliable inter-laboratory comparison in foodborne pathogen surveillance.

## 1 Introduction

In recent years, public health, food safety and veterinary laboratories transitioned to the application of whole-genome sequencing (WGS) for molecular surveillance in food-related outbreak investigations, offering a higher precision compared with older techniques, such as pulsed-field gel electrophoresis. With the application of WGS, extensive genetic information regarding the sequence type and phylogenetic relationship is obtained, together with additional information on antimicrobial resistance profiles, virulence factors and mobile genetic elements (Balloux et al., 2018). The detection of clusters of genetically highly similar bacterial strains, is a major task for retrospective and prospective routine outbreak investigations, as well as for source attribution (EFSA Panel on Biological Hazards et al., 2019).

The initial steps aiming at WGS-based cluster identification can be divided into two fundamental parts, the wet-lab part including the DNA library preparation and sequencing process, and the subsequent dry-lab part including bioinformatic analyses and data interpretation. Arguably, one of the greatest challenges in outbreak analysis was the lack of standardization in both parts. Varying approaches or even small deviations in library preparation, sequencing and bioinformatic analysis impacted the overall result, namely whether the isolate under consideration clustered with potentially outbreak-related isolates or not. General requirements for genomic characterization of food-borne bacterial pathogens have been defined (International Organization for Standardization, 2022). Hence, many German food-safety laboratories in the federal states transitioned to short-read sequencing for outbreak analysis in the last decade, adopting protocols, instruments and data analysis software according to their tasks and needs in the absence of a prescribed standardized procedure. In 2022, an official German-wide investigation procedure for sequencing of Shiga toxin-producing *Escherichia coli* (*E. coli*), *Listeria monocytogenes* (*L. monocytogenes*), *Salmonella enterica* (*S. enterica*), and *Campylobacter jejuni/C. coli* (*Campylobacter* spp.) was developed and approved by a working group of the German Federal Office of Consumer Protection and Food Safety as part of the § 64 German Food and Feed Code (LFGB). This working group brought together stakeholders and expertise of federal state laboratories, federal institutes, companies and universities to define a common ground for high-quality data usable for outbreak analysis (Szabo et al., 2020). In a recent interlaboratory study, we analyzed the impact of different wet-lab protocols on sequence quality, based on 10 isolates for each of the four food-related species. We showed that, while the overall sequence quality was high and mostly comparable, application of GC-biased library preparation kits or sequencing instruments by different manufacturers impacted the quality of raw sequence data (Uelze et al., 2020a; Forth et al., 2023). Based on these experiences, we defined detailed wet-lab protocols for bacterial WGS (Bundesamt für Verbraucherschutz und Lebensmittelsicherheit, 2023).

Transforming large amounts of sequencing data into information involves several bioinformatic analysis steps and relies on substantial computing resources and infrastructure. An abundance of tools exists for genome assembly, microbial typing and sequence comparison. These tools are diverse; for example, they can be designed for Windows or Linux operating systems, are command-line based or have a graphical user interface, are open-source or commercial software, and may be aggregated into pipelines or software suites (Jünemann et al., 2014; Schürch et al., 2018; Uelze et al., 2020b). Naturally, these methods differ in their output (Mollerup et al., 2022). As software may contain several layers (e.g., Shovill^1^ as a wrapper for different assembly algorithms) and the output of one tool can serve as the input for the next (e.g., assembled genomes as a basis for allele calling), variations accumulate in the final result and are difficult to pin down to its origin.

Similar to the wet-lab procedures, German federal state laboratories use different software tools for similar tasks, due to differences in bioinformatic capabilities and expertise in individual teams. In the above-mentioned working group, a guideline has been developed describing necessary steps of bioinformatic analysis and best-practices to ensure high-quality analyses (Bundesamt für Verbraucherschutz und Lebensmittelsicherheit, 2024). In this framework, we conducted a dry-lab inter-laboratory study, focusing on the bioinformatic analysis of four bacterial species to uncover potential variability introduced by differing data analysis approaches and human interpretation. Participants were asked to apply their routine in-house protocols while following the general rules of the guideline. Steps included a quality assessment (and sample exclusion), 7-gene Multilocus-Sequence Typing (MLST), core genome Multilocus Sequencing Typing (cgMLST), and Single Nucleotide Polymorphism (SNP) calling. Subsequently, participants were asked to identify clusters. The scope of the study was not to resemble a proficiency test with a final pass/fail result but to assess and quantify variability in the results and, where possible, identify the underlying reasons for such variation. For this purpose, the datasets also included border-line raw data in terms of quality.

## 2 Material and methods

### 2.1 Implementation of the inter-laboratory study

#### 2.1.1 General design

The inter-laboratory study was jointly organized by the German Federal Institute for Risk Assessment and the German Federal Institute for Animal Health -Friedrich-Loeffler-Institut, and carried out in autumn 2023. Seventeen participants from federal state laboratories, federal institutes and one university were enrolled. One participant withdrew mid-study due to staff shortage. The time frame for conducting the bioinformatic analyses was initially set to 5 weeks followed by a 3-week extension. Initially, laboratories received an anonymized laboratory code and a link to a password-protected cloud-based platform *via* email, where all necessary files to conduct the inter-laboratory study were available for download: the guideline for cluster analysis, the protocol detailing the instructions for analysis, the raw sequencing dataset including MD5-checksums, a sample sheet for sample name-to-data-allocation, a reference genome for SNP calling and template data sheets (one per target species) for the results. The laboratories were asked to perform their routinely applied bioinformatics workflows for data analysis and evaluation. Participants were given the option to analyse the datasets according to their expertise regarding the species, not necessarily processing all four datasets. Additionally, they were encouraged to submit more than one result file, if they applied different bioinformatic workflows/pipelines/schemes to a data set. The result file(s) were then transmitted to the organizers *via* email for data aggregation and evaluation.

#### 2.1.2 Provided data

For each of the four species, the dataset consisted of raw Illumina paired-end sequencing data of 50 isolates (available *via* https://zenodo.org/records/14006501). The datasets for each species purposely included 10 % raw sequencing data with quality issues including low coverage, low quality and contaminated data. Participants were asked to identify and exclude these data according to quality criteria described in the German official collection of test methods (Bundesamt für Verbraucherschutz und Lebensmittelsicherheit, 2023; Table 1). Raw and unmodified sequencing data was used with the exception of two *Campylobacter* samples: (i) for Camp_48 the raw sequencing reads of two different *C. jejuni* samples were mixed to simulate inter-species contamination, (ii) Camp_49 was a mix of raw sequencing reads from *C. jejuni* and *S. enterica*. The rationale of the *in-silico* contamination data of *Campylobacter* strains was the absence of real-contamination data in the original data set.

**Table 1:** Quality thresholds for exclusion of provided raw reads samples from downstream analysis according (Bundesamt für Verbraucherschutz und Lebensmittelsicherheit, 2023).

**Quality metrics**	***Salmonella enterica***	***Listeria monocytogenes***	***Campylobacter coli/C. jejuni***	***Escherichia coli***
% of bases with >Q30 (2x 150bp)	80	80	80	80
Depth of coverage	>30 and < 200	>20 and < 200	>20 and < 200	>40 and < 200
Inferred genus	*Salmonella*	*Listeria*	*Campylobacter*	*Escherichia/Shigella*
% of reads not assigned to target genus	< 10	< 10	< 10	< 10
Total length of the assembly (in Mb)	4.3–5.2	2.7–3.2	1.5–1.9	4.5–5.9
Number of contigs	≤ 300	≤ 300	≤ 300	≤ 500
% GC content before and after assembly	52.1 ± 4	37.9 ± 4	31.3± 4/30.4 ± 4	52.1 ± 4

The data was selected randomly without reference to former outbreaks and provided without epidemiological metadata. Given the task was to perform a cluster analysis without drawing epidemiological conclusions regarding an outbreak situation, isolates of interest were termed “focus isolates” and the corresponding clusters “focus clusters,” to avoid association with the term “outbreak.”

#### 2.1.3 Detailed instructions

**Figure 1:**
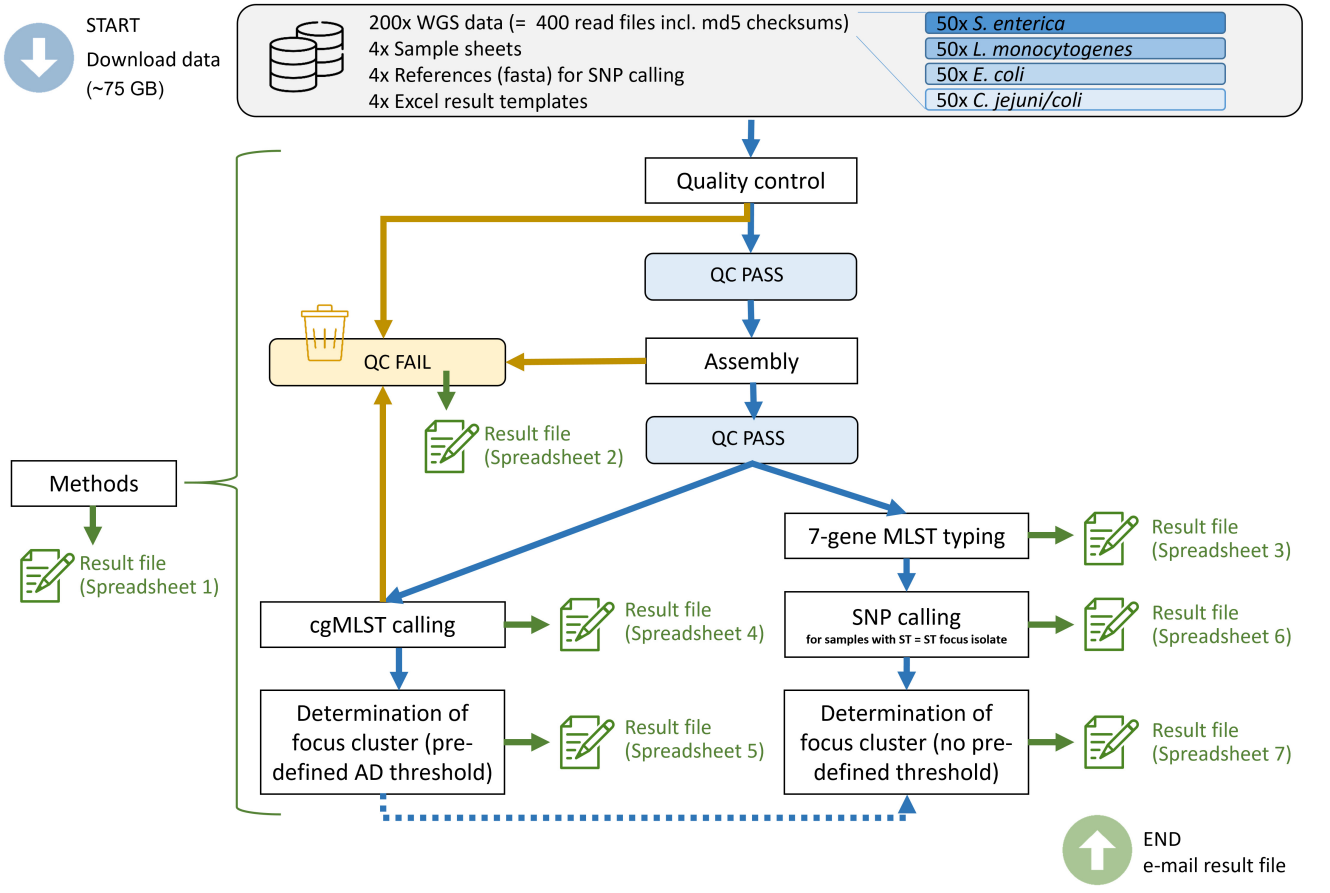
Overview of the inter-laboratory study design and the steps conducted by the participants. WGS, whole-genome sequencing; QC, quality control; AD, allelic distance.

We provided instructions on how to conduct the inter-laboratory study within the framework of the cluster analysis guideline (Figure 1). In summary, the participants had to perform the following steps: quality control and exclusion of samples with low quality according to the given criteria (Bundesamt für Verbraucherschutz und Lebensmittelsicherheit, 2024), assembly, MLST typing, cgMLST calling with focus cluster determination (predefined threshold values) and SNP calling with focus cluster determination (without a given threshold). The results of each participant were collected in a MS-Excel file consisting of seven predefined spreadsheets: (1) A questionnaire with 82 questions on details of the applied methods, schemas, deviations from the default parameters etc.; (2) a list of excluded samples due to low quality and the reason for exclusion; (3) 7-gene MLST typing results; (4) cgMLST distance matrix; (5) samples with pre-defined AD cut-off to the focus strain (cluster analysis based on cgMLST); (6) SNP distance matrix; (7) focus cluster determination based on SNP analysis (isolates in a cluster with the focus strain, no pre-defined SNP cut-off).

Details on quality control and assembly: For assembly, software had to be applied that is based directly or indirectly on the algorithms of SPAdes^2^ in at least version 3.14 or SKESA^3^ with version 2.4.0 or higher. The quality of the raw data and assemblies should be assessed based on the mandatory and optional quality criteria of the German official collection of test methods (§ 64 German Food and Feed Code) with regard to the percentage of bases with >Q30 sequencing quality, depth of coverage, contamination control, total length of the assembly, inferred genus, number of contigs and GC content before and after assembly (Bundesamt für Verbraucherschutz und Lebensmittelsicherheit, 2023; Table 1). Data sets that did not pass the initial quality control had to be excluded from further analysis by the participants. Reasons for exclusion of samples were collected in spreadsheet 2 of the result file.

Based on assemblies of sufficient quality, participants were asked to perform 7-gene MLST and to enter sequence types (STs) in spreadsheet 3. Participants were free to select software and schemas, provided that the scheme was internationally available and acknowledged by the scientific community.

Based on assemblies of sufficient quality, cgMLST cluster analysis had to be performed, with the algorithm and schema freely selectable with the same restrictions as for 7-gene MLST. Participants were asked to add assemblies with an insufficient number of loci (≥5% missing loci) in the list of data with insufficient qualities of spreadsheet 2 and excluded from further analyses. The cgMLST distance matrix was collected (spreadsheet 4), as well as a list of all closely related isolates in the respective “focus cluster,” in spreadsheet 5 using 10 allelic distances (ADs) as threshold (7 AD for *L. monocytogenes*).

Genotyping based on Single Nucleotide Polymorphisms (SNPs) using the provided reference genomes (Table 2) had to be performed on the raw reads of all samples that were of the same Sequence Type (ST) as the focus strain and that were not excluded due to low quality in previous analyses. The SNP distance matrix was collected (spreadsheet 6) as well as a list containing samples within the focus cluster and their number of high-quality SNPs (no predefined cut-off for focus cluster determination; spreadsheet 7).

**Table 2:** Specified reference for SNP analysis and given Allelic Distances (AD) threshold for cgMLST cluster definition.

**Species**	**Reference**	**AD cluster threshold cgMLST**
*Campylobacter coli/C. jejuni*	NZ_CP007192.1.fasta	10
*Escherichia coli*	NZ_CP008957.1.fasta	10
*Listeria monocytogenes*	NZ_CP028183.1.fasta	7
*Salmonella enterica*	NZ_CP019649.1.fasta	10

### 2.2 Data aggregation and evaluation

The transmitted results per spreadsheet and species were collected and non-contentious divergences from the predefined templates (e.g., typos, misplacing of cells in tables) were corrected. Exploratory data analysis and visualization (e.g., alluvial flows, bar plots, dot plots) were performed using the statistical language R (R Core Team, 2022).

To compare all possible clusters (not only the focus cluster) detectable by the different analysis approaches, hierarchical clustering with single linkage was performed based on the distance matrixes provided by the participants. The clustering threshold for cgMLST was defined to 10 AD (7 AD for *L. monocytogenes*) by the organizers. Adjusted Rand indices were calculated using R's fossil package and visualized using pheatmap.^4^ For SNP calling, thresholds of 10 (*L. monocytogenes, S. enterica*), 15 (*E. coli*), and 40 (*C. jejuni*) SNPs were used, respectively.

## 3 Results

In this inter-laboratory study, 16 of 17 participants transmitted one to eight result tables, depending on the number of species analyzed and methods/schemas used. Participants were encouraged to analyze the same data set with different combinations of tools, schemas or settings. Each submitted results table contained one analysis approach (combination of tools and schemas), a term that will be used from here on. Overall, we received 75 results files by 16 participants, which distributed into 20 files of 13 participants for *Campylobacter* data, 19 files of 12 participants for both *E. coli* and *L. monocytogenes* data, and 17 files of 12 participants for *S. enterica* data.

### 3.1 Applied tools and schemas

**Figure 2:**
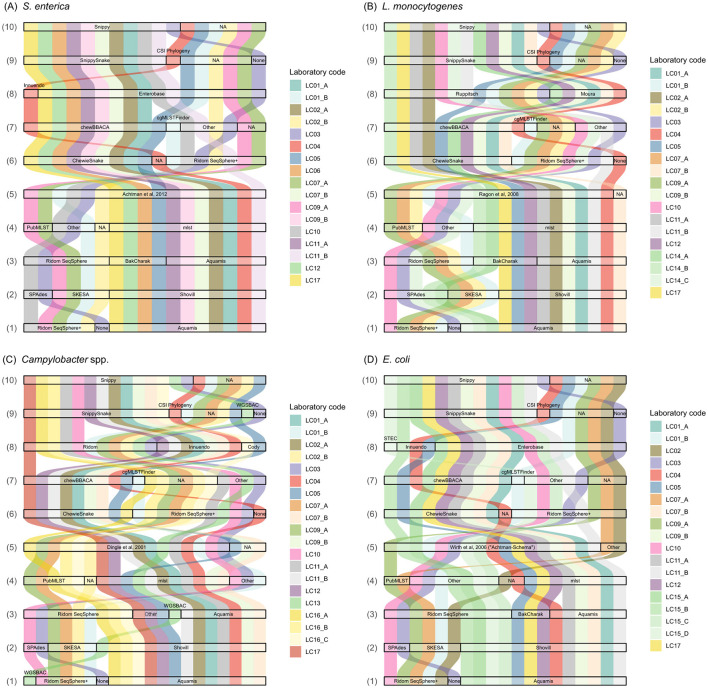
Selection of applied tools and schemas in the analysis of **(A)** *S. enterica*, **(B)** *L. monocytogenes*, **(C)** *C. jejuni*, and **(D)** *E. coli* sequence data. (1) Assembly & quality control software/pipeline, (2) Assembly tool, (3) 7-gene MLST pipeline, (4) 7-gene MLST tool, (5) 7-gene MLST scheme, (6) cgMLST software/pipeline, (7) cgMLST tool, (8) cgMLST scheme, (9) SNP analysis software/pipeline, (10) SNP analysis tool. NA, not available.

Overall, 82 questions were answered by the participants regarding their analysis approach(es) on the quality control, assembly, 7-gene MLST typing, cgMLST and SNP calling. The questions and answers on tools, versions, (cg)MLST schemas, deviations from the default settings etc., were assembled in Supplementary Table 1. An overview of selected tools and schemas by the participants is presented in Figure 2.

For assembly and quality control, the majority of analysis approaches applied the software suite Ridom SeqSphere+^5^ or the pipeline AQUAMIS (Deneke et al., 2021a; Figure 2). For example, out of 17 submitted analysis approaches applied to the *S. enterica* data set, AQUAMIS was used 10 times (58%) and Ridom SeqSphere+ five times (29%). One participant (LC03) applied SPAdes directly. For *Campylobacter* spp. assembly and quality control, LC13 applied the pipeline WGSBAC (El-Adawy et al., 2023). Both pipelines AQUAMIS and WGSBAC comprise Shovill, footnote 1 that can be run with the SPAdes or SKESA assembly algorithms. With the exception of LC09_B, all participants applying Shovill opted for SPAdes as the assembler. In contrast, out of five participants employing Ridom SeqSphere+, four opted for SKESA and one for SPAdes as the assembler, independent of the species investigated (Figure 2).

Sequence types of 7-gene MLST were determined using either the software suite RidomSeqSphere+ or the automated pipelines BakCharak,^6^ AQUAMIS or WGSBAC (Figure 2). While the three pipelines are all based on the tool mlst,^7^ participants who carried out sequence typing in RidomSeqSphere+ mainly applied the online version of the tool at PubMLST (Jolley et al., 2018). The applied typing schemas were consistent across all analysis approaches using Achtman et al. (2012) for *S. enterica*; Ragon et al. (2008) for *L. monocytogenes*; and Dingle et al. (2001) for *C. jejuni*. Three participants presumedly forgot to enter the applied scheme in the questionnaire, however, based on the typing results the scheme could be deduced. For *E. coli*, all analysis approaches were based on the scheme by Wirth et al. (2006) with two analysis approaches providing additional information by typing with the Pasteur scheme (BIGSdb; Jaureguy et al., 2008).

Core-genome MLST was exclusively performed either with the pipeline ChewieSnake (Deneke et al., 2021b) or within the software suite Ridom SeqSphere+, with the exception of participant LC04 who preferred to apply the tool chewBBACA (Silva et al., 2018) directly (Figure 2). In detail, nine out of 17 analysis approaches (52%) for *S. enterica* used ChewieSnake, while seven (41%) used Ridom SeqSphere+. Regarding the applied scheme, for *S. enterica*, 16/17 (94%) analysis approaches applied the Enterobase scheme (Zhou et al., 2020). For *L. monocytogenes*, 13/19 (68%) analysis approaches applied the Ruppitsch scheme (Ruppitsch et al., 2015) and in 6/19 (31%) cases the Moura scheme (Moura et al., 2016) was used. For *C. jejuni*, 11 approaches (57%) chose the cgMLST Ridom SeqSphere+ scheme^8^ (637 loci), six approaches (31%) the 678 loci Innuendo scheme (Rossi et al., 2018a; Nennig et al., 2020), two the Cody scheme (10%, 1343 gene loci; Cody et al., 2017) and one selected “other” and specified “chewBBACA online” (Mamede et al., 2021). For *E. coli*, the majority (15/19, 78%) was based on the Enterobase scheme (Zhou et al., 2020) with 3/19 (16%) based on the Innuendo scheme (Rossi et al., 2018b) and one approach based on a specific STEC scheme^9^ used in combination with Ridom SeqSphere+ (Figure 2).

For SNP calling, the pipelines SnippySnake^10^ (Lüth et al., 2021), CSI Phylogeny (Kaas et al., 2014) and WGSBAC (El-Adawy et al., 2023) were applied, with the majority of analysis approaches focusing on SnippySnake. Both, SnippySnake and WGSBAC rely on Snippy^11^ for SNP-calling and construction of a core-genome. In one analysis approach (LC03) the tool Snippy was applied directly. While all participants provided SNP results, some participants provided several results sheets with differing analysis approaches (e.g., differing in cgMLST schemas). However, this did not affect SNP results and therefore, duplicated SNP results were not considered (depicted as NA in Figure 2). LC03 performed SNP analysis including all high-quality samples for the four species, respectively, instead of focusing on samples sharing the same ST as the focus strain.

### 3.2 Quality control

The quality of the raw data and assemblies was assessed based on the mandatory and optional quality criteria of the German official collection of test methods (Bundesamt für Verbraucherschutz und Lebensmittelsicherheit, 2023; Table 1). Participants reported which data set was removed from further analysis due to insufficient quality including the reason(s) why the specific data set was excluded (Supplementary Table 2). For *L. monocytogenes*, users excluded between one and nine samples. Sample List03 was excluded by all analysis approaches for reasons including intra-species contamination, large number of contigs and large number of missing cgMLST loci. For the same reasons List01, List04, and List05 were excluded by more than 50% of analysis approaches. Insufficient Q30 values were reported for List23 by 14 (73%) analysis approaches. Up to eight analysis approaches reported low quality for additional samples mainly due to potential intra-species contamination.

A set of five samples was excluded by almost all (out of 20) analysis approaches for *C. jejuni*: Camp01 (*n* = 19) as it was the wrong species (*C. fetus*), Camp46 and Camp47 (both *n* = 19) due to low coverage, Camp48 (*n* = 20) and Camp49 (*n* = 19) because of contamination. Four analysis approaches excluded up to 28 samples mainly due to a low number of called cgMLST loci. Those users probably applied a threshold of 5% missing loci, while other users used 10%. Up to seven additional samples were excluded by a small number of analysis approaches mainly based on intra-species contamination.

As far as *E. coli* isolates were concerned, the samples Ecoli08 and Ecoli40 were excluded by all analysis approaches due to low coverage as well as intra-species contamination (only Ecoli40). Twelve (out of 18, 66%) analysis approaches removed Ecoli12 and Ecoli20 from further analysis as they detected inter-species contamination, while Ecoli31 was removed by 11 participants due to intra-species contamination.

Two *S. enterica* data sets were removed in all submitted analysis approaches: Salm49, because the assembly length was too short and Salm50 due to inter-species contamination. Fourteen (out of 17, 82%) of the analysis approaches excluded Salm48 due to too many missing loci for cgMLST. Salm29 was removed from nine (52%) reported results as participants detected intra-species contamination. For the same reason, some users also excluded Salm11 (*n* = 2), Salm43 (*n* =1) and Salm44 (*n* = 1).

### 3.3 Multilocus sequence typing

Participants were asked to perform MLST on those assemblies they did not exclude due to failure in the previous quality control step. As the sample quality was judged differently depending on analysis approach, different numbers of samples were included in the MLST analysis. The majority of typing results for assemblies based on high-quality sequence data were congruent (Supplementary Table 3). In fact, for all assemblies derived from high-quality *C. jejuni/C. coli* and *S. enterica* data, ST results were identical. For *E. coli*, the analysis was based on the “Achtman” scheme (Zhou et al., 2020), where all participants detected the same STs, with the exception of one assembly, which was not typable in spite of high-quality sequence data (LC03 – Ecoli47). Two participants (LC02, LC07) provided additional typing results using the “Pasteur” scheme (Jaureguy et al., 2008), which indicated the same grouping of samples as achieved with the “Achtman” scheme. For three high-quality *L. monocytogenes* samples, the ST results of two participants differed from those of all other participants: LC03 detected ST121 instead of ST5 for samples List36 and List37, LC04 determined ST5 instead of ST9 for List48. In some cases, participants performed MLST on low-quality assemblies, contrary to the instructions. In such cases, ST results were sometimes inconsistent among participants (e.g., for Camp49 with a strong inter- and intra-species contamination, LC03 reported ST572 compared to ST4 reported by LC12).

### 3.4 Core-genome multilocus sequence typing

Participants were asked to perform cgMLST based on high-quality assemblies. Using the retrieved allelic differences, participants were asked to provide a list of samples that “cluster” with the focus isolate using 10 AD as the threshold (7 AD for *L. monocytogenes*). A small number of participants (usually one to two per species) misunderstood the instructions and provided a list of the AD of all samples to the focus isolate, thus not providing clustering interpretation. In these cases, we applied the AD thresholds to the provided AD matrices and determined the lists of samples clustering with the focus isolate.

**Figure 3:**
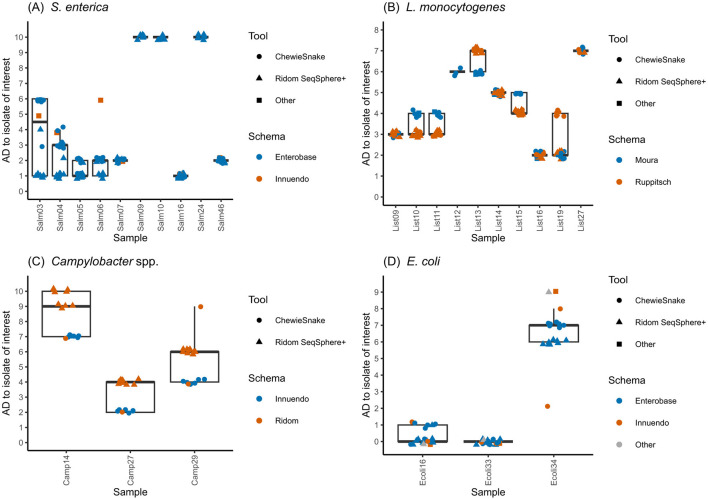
Results of cgMLST. Allelic distances of strains clustering with the focus strain, comparing different schemas and tools for **(A)** *S. enterica*, **(B)** *L. monocytogenes*, **(C)** *C. jejuni*, and **(D)** *E. coli* sequence data. Individual analyses are named according to the following format: LC_approach_pipeline_assembler_cgMLST-tool_scheme; LC, Laboratory Code (“_A,”… indicates different analysis approaches), Pipelines: Rs, Ridom Seqsphere+; Wg, WGSBAC; Aq, AQUAMIS; Assembler: Sh, Shovill; Sp, Spades; Sk, Skesa; cgMLST tools: Rs, Ridom SeqSphere+; Cs, Chewiesnake; NO, none; Schemas: Eb, Enterobase; Cd, Cody; Rs, Ridom SeqSphere+; In, Innuendo; Mo, Moura; Ru, Ruppitsch; St, STEC.

With the exception of LC01_A, all analysis approaches that were carried out using *S. enterica* assemblies included either seven (using ChewieSnake) or 10 (using RidomSeqSphere+) strains in a cluster with the focus strain (Salm15, Supplementary Figure 1A, Supplementary Table 4). The three deviating samples (Salm09, Salm10 and Salm24) had AD values near the cut-off of 10 AD (exactly 10 AD in the RidomSeqSphere+ analysis and 11-12 AD in the ChewieSnake analysis) and were therefore inconsistently included in or excluded from the focus cluster. Generally, analyses employing ChewieSnake tended to yield higher ADs to the focus isolate than analyses with RidomSeqSphere+ (Figure 3A). However, for three samples (Salm 07, Salm16 and Salm46), reported ADs were identical, irrespective of the analysis tool used. The largest difference (5 ADs) was observed for Salm03.

Regarding *L*. *monocytogenes*, data analysis the number of samples reported to cluster with the focus isolate (List20) varied between six and 10 (Supplementary Figure 1B). These differences were mainly attributed to different decisions regarding the exclusion of low-quality samples (Supplementary Table 4). For example, the strains List09, List12, and List13 were excluded by some analysis approaches due to potential intra-species contamination. Seven strains were reported to be part of the focus cluster by all 19 analysis approaches, irrespective of the applied assembler, allele-caller and scheme (Figure 3B). List27 was the only strain not excluded for quality reasons, but was reported only by eight analysis approaches to cluster with the focus strain. A detailed analysis of all 10 strains reported as clustering with the focus isolate revealed a maximum difference of two ADs when comparing different assemblers, allele-callers and schemas.

Using different cgMLST schemas with different resolution, participating laboratories obtained different results. 16 analysis approaches clustered Camp14, Camp27, and Camp29 together with the focus strain (Camp28), using the lower resolution cgMLST schemas of Ridom and Innuendo with 637 and 678 gene loci, respectively (Figure 3C, Supplementary Figure 1C, Supplementary Table 4). One approach (LC17) did not report Camp14. Repetitive analysis with Ridom SeqSphere+ consistently produced the same ADs of the three strains to the focus strain. The same was true for ChewieSnake, although two different schemas were used here. The tool Ridom SeqSphere+ was only combined with the Ridom SeqSphere+ scheme,^12^ and produced consistently larger ADs (maximum of three) than analyses performed with ChewieSnake in combination with either Innuendo-scheme or Ridom SeqSphere+ scheme. Three analysis approaches did not report any strain clustering with the focus strain: LC05 and LC16_C applied the higher resolution Cody scheme (1,343 gene loci) and reported larger AD of Camp14, Camp27, and Camp29 to the selected focus strain ranging from 15 to 22 AD. LC04 reported using “Innuendo online” (Rossi et al., 2018a) and also came to the conclusion that Camp28 did not cluster with any other sequence. Moreover, LC16 reported another cluster containing the strains Camp04 with Camp02 and Camp05 with AD < =2 using the cgMLST Cody and AD=0 using the Ridom scheme.

*E. coli* focus clusters submitted by the participants consisted of four samples (including the focus isolate), which were reported in all analysis approaches, irrespective of the assemblers, cgMLST pipelines and schemas (Figure 3D, Supplementary Figure 1D) that were used. All analysis approaches, irrespective of tool and scheme, resulted in zero AD to the focus isolate Ecoli33 and one to two ADs for Ecoli16 (Supplementary Table 4). For Ecoli34, most analyses consistently reported six ADs (Ridom SeqSphere+) and seven ADs (ChewieSnake) when using the Enterobase scheme, whereas analyses using the Innuendo or STEC schemas footnote 9 (LC15_D) reported nine ADs.

In addition to the analysis of focus clusters where participants detected clusters of predefined focus strains, we used the reported complete cgMLST-distance matrices to perform hierarchical clustering. With this, we aimed to compare all clusters generated by the different approaches, instead of focusing on only the focus cluster. To compare the different partitions, we calculated adjusted Rand indices and compared all clusters that were detected. For *S. enterica*, this analysis resulted in *three* clusters. Adjusted Rand indices ranged between 0.9 and 1. Here, the choice of the cgMLST pipeline (Ridom SeqSphere+ vs. ChewieSnake) had a slightly higher influence on Rand indices than the choice of the scheme (Supplementary Figure 3A). For *L. monocytogenes*, hierarchical clustering yielded the same eight clusters for all analysis approaches (Supplementary Figure 13B). The adjusted Rand index for cluster accordance ranged between 0.82 and 0.99. The use of different schemas (Moura vs. Ruppitsch) caused slightly more differences than the use of different pipelines (Ridom SeqSphere+ vs. ChewieSnake). For *Campylobacter*, 16 analysis approaches used lower resolution cgMLST schemas of Ridom and Innuendo. Between two and three clusters were detected and the adjusted Rand indices ranged between 0.84 to 1 (Supplementary Figure 3C). Here, applying different pipelines (Ridom SeqSphere + vs. ChewieSnake) caused larger differences in the clustering results compared to applying different schemas (Ridom SeqSphere + vs. Innuendo). Three analysis approaches applied higher resolution schemas. LC05 and LC16_D both applied the Cody scheme, whereas LC04 selected “Innuendo” in the predefined results table. The two approaches using the Cody scheme produced the exact same clustering results indicated by an adjusted Rand index of 1. Rand indices below 0.75 were achieved comparing low with high resolution cgMLST schemas. For *E. coli*, three clusters were detected, each containing three to four strains. The adjusted Rand index was 1, except for LC15_D (0.94; Supplementary Figure 3D).

### 3.5 Single nucleotide polymorphism based typing

All analyses performed by the participants were based on the provided reference sequences (Table 2). All participants only included isolates with the same ST as the focus strain, in accordance with the instructions, except for LC03, who performed SNP typing with all respective strains. Since the addition of isolates from different STs affects the core genome derived for SNP calling, the results of LC03 were excluded for this part of the analysis. In addition, participants were asked to list samples that fall within the focus cluster. In contrast to the instructions for cgMLST analysis, no predefined threshold was defined for SNP calling. Therefore, each participant applied an individual threshold, determined isolates belonging to the focus cluster and listed the number of detected SNPs with respect to the focus strain for those samples. Two participants did not define a focus cluster and erroneously listed the number of SNPs for all samples (LC01_A for all species and LC14_A for *L. monocytogenes*). In these cases, only the samples that were congruent with the focus clusters and thresholds determined by most of the analysis approaches were compared to other participants' results.

**Figure 4:**
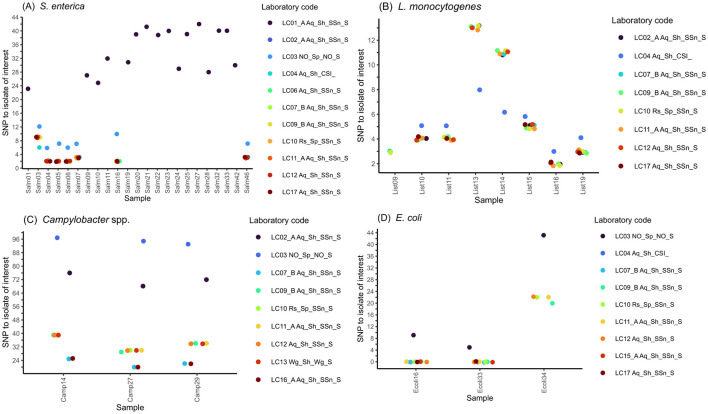
Results of SNP-typing. SNP distances of strains clustering with the focus strain, comparing different SNP callers for **(A)** *S. enterica*, **(B)** *L. monocytogenes*, **(C)** *C. jejuni* and **(D)** *E. coli* sequence data. Individual analyses are named according to the following format: LC_approach_SNP-pipeline_SNP-tool; Pipeline: SSn, SnippySnake; Wg, WGSBAC; CSI, CSI Phylogeny; SNP tool: S, Snippy.

Regarding *S. enterica* data, each of the 10 analysis approaches clustered a set of seven strains together with the focus strain (Figure 4A, Supplementary Figure 2A). The same cluster was detected with cgMLST using ChewieSnake (Figure 3A). The number of SNPs called was identical between Snippy/SnippySnake and CSI Phylogeny (used in one analysis approach) results with the exception of sample Salm03, where Snippy identified three additional SNPs compared to CSI Phylogeny.

For *L. monocytogenes*, the participants included five to eight strains clustering with the focus isolate (List20): List10, List11, List15, List16, and List19 were present in all reported clusters (Figure 4B, Supplementary Figure 2A). List13 and List14 were also included by most analysis approaches, except for the one employed by LC17A (Supplementary Table 5). In addition, List09 was included by four approaches. Quality metrics below the defined quality thresholds were the reason for exclusion for most of the samples that were not included in the focus cluster. The number of called SNPs was identical among analyses that relied on SnippySnake (employed by nine analysis approaches). Using CSI phylogeny (one participant), the analysis differed by one additional SNP for five samples and five less SNPs for two samples. All eight strains forming a SNP cluster with the focus strain were also included by the cgMLST analyses, although some approaches added two additional strains (Figure 3A) to the cluster.

For *C. jejuni*, eight participants listed three samples (Camp14, Camp27, Camp29) in the focus cluster (Figure 4C, Supplementary Figure 2C). LC01A additionally listed Camp10 (909 SNPs), LC17 stated that there are no samples clustering with the focus strain, and LC04 did not fill in the list (unclear if by mistake). The clustering of Camp14, Camp27, and Camp29 were consistent with results of the cgMLST analysis using the Ridom SeqSphere+ or Innuendo schemas (Figure 3C). The number of detected high-quality SNPs varied between those analyses that applied a Gubbins filter (20-24 SNPs) and those that did not (30-40 SNPs). For LC02, the number of SNPs compared to the focus strain varied between 68 and 76.

The SNP-based focus cluster for *E. coli* contained three samples (Supplementary Table 5, Supplementary Figure 2D), of which two samples (Ecoli16 and Ecoli33) were identified by all nine analysis approaches and showed zero SNPs using SnippySnake (Figure 4D). One sample (Ecoli34), comprised a larger number of SNPs (>20), and was included in the focus cluster by five out of nine participants. The inclusion of Ecoli16, Ecoli33 and Ecoli34 was consistent with all analysis approaches using cgMLST (Figure 3D).

Hierarchal clustering using the participants' SNP-matrices, yielded very similar clusters for *S. enterica* indicated by adjusted Rand indices ranging from 0.99 to 1 (Supplementary Figure 4). For *L. monocyotogenes*, each analysis approach detected eight clusters. The adjusted Rand index for repeated analysis based on Snippy (different versions and settings) ranged from 0.97 to 1, while it was slightly lower comparing CSI Phylogeny to Snippy. For *E. coli*, each analysis approach detected the same cluster while for *C. jejuni* different partitions were created. Analysis approaches that used Snippy (WGSBAC, SnippySnake) and did not apply a recombination filter detected the exact same clusters. The four approaches (LC04, LC07_B, LC16_A and LC17) using the optional recombination filter also produced the same clusters when compared with each other

However, different partitions were created comparing analysis approaches with or without recombination filter indicated by a Rand index of 0.55. As LC02 reported more than 68 SNPs compared to the focus strain, no cluster was detected in Rand analysis.

## 4 Discussion

Whole-genome sequencing of foodborne bacterial pathogens has become routine for analysis and detection of outbreaks worldwide (Food and Agriculture Organization of the United Nations, 2016; Allard et al., 2018; Stevens et al., 2022). However, standardization and training are needed for both, wet-lab and dry-lab data analysis. In Germany, food safety is the legal obligation of the laboratories of 16 federal states. A working group “NGS-Bacterial-Characterization” organized by the German Federal Office of Consumer Protection and Food Safety brings together expertise of federal state laboratories, federal institutes, companies and universities aiming at harmonization and training of bacterial WGS (Szabo et al., 2020). So far, standardized wet-lab protocols have been developed and evaluated (Bundesamt für Verbraucherschutz und Lebensmittelsicherheit, 2023; Forth et al., 2023). Given that various data analysis tools and settings may reveal different outbreak clusters, a guideline explaining the essential steps for bioinformatic analysis and best practices to achieve high-quality clusters was then published by the German working group (Bundesamt für Verbraucherschutz und Lebensmittelsicherheit, 2024). In line with this guideline, this study conducted an inter-laboratory dry-lab investigation with an emphasis on bioinformatic analysis to identify any potential variability in cluster detection brought about by various data processing techniques and human interpretation. Participants were requested to adhere to the guidelines. Wherever the guidelines allowed for different options (e.g., MLST schema) participants were asked to carry out their regular gMLST practices.

In this inter-laboratory study, we aimed for a uniform procedure applicable to the four most relevant foodborne pathogenic species investigated. The approaches for cluster analysis in a real outbreak investigation may differ depending on the pathogen (e.g., inclusion of serotypes, *stx* genes, threshold, etc.). However, the general steps applied in this study are most likely to be recognized in any of the individual approaches, although they may differ in their selection of samples for subsequent analyses.

Each participant was asked to complete a questionnaire for in-depth description of applied methods and results. This included, among others, names and version of applied pipelines, analysis tools, (cg)MLST schemas and parameter settings (other than standard). In a few cases, fields were not, or wrongly, filled in (as deduced by the provided results). For example, six different answers were given regarding the allele-caller of the Ridom SeqSphere+ software suite.

The analysis approaches selected by the participants were quite diverse in terms of applied tools, (cg)MLST schemas and settings. However, compared with the number of tools available, it represents a relatively small fraction, with general trends being clearly visible: the focus on the Ridom SeqSphere+ software suite and on the pipelines AQUAMIS, ChewieSnake and SnippySnake. In general, results generated by pipelines are more reproducible than those generated by the manual application of tools, given that human error is minimized and settings and parameters are clearly defined and can be recalled easily.

In spite of pre-defined quality criteria (Bundesamt für Verbraucherschutz und Lebensmittelsicherheit, 2023; Table 1), the assessment of sample quality and decision on inclusion for further analysis was highly variable. We assume this to be due to varying levels of user experience and interpretation of quality metrics. Also, some pipelines (e.g., AQUAMIS) provide more tools for quality assessment than others (e.g., Ridom SeqSphere+) such that the choice of pipeline also impacts the repertoire for quality assessment. Additionally, the application of some quality criteria was optional and/or included non-stringent threshold ranges. For example, the protocol included in this study only suggests to check for contamination without providing specific thresholds for sample exclusion, leading to differences in interpretation. The results of the inter-laboratory study showed the full spectrum of quality checks applied by the participants — from very rigorous quality control (exclusion at the point of slight deviations from the optimum) to less-stringent, and even insufficient, quality checks. However, only in rare cases, raw sequence data with a very low Q30 base fraction were not excluded. The participants' decision on including samples especially varied for samples with quality measures in the “gray” area. Here, additional detailed data provided by the applied pipelines can be used to determine quality. For example, contamination is often checked by classifying reads (or contigs) using Kraken2 (Wood and Salzberg, 2014; Wood et al., 2019). However, Kraken2 may “misclassify” sequences from mobile genetic elements (e.g., plasmids). As the existence (and number) of plasmids is predicted by most pipelines, users may check this information in order to understand if the Kraken results are based on a real contamination. A large number of samples was excluded because of intra-species contamination predicted by the tool conFindR (Low et al., 2019). This tool reports the presence of single nucleotide variants in a predefined set of core, single-copy loci. The conFindR with its default thresholds is very sensitive in detecting small intra-species contaminations that frequently occur on Illumina devices (e.g., carry-over contaminations). However, participants had difficulties to define or use thresholds to exclude samples based on intra-species contamination. Excluding isolates obviously may impact cluster definitions. However, including potentially intra-species contaminated samples can confound clustering analyses based on SNP and cgMLST typing methods (Pightling et al., 2019). In summary, laboratory experience, access to bioinformatic tools as well as communication of thresholds turn out to be key for a concise quality assessment.

The majority of 7-gene MLST typing results was congruent among all participants, with very few exceptions. In some cases, contrary to the instructions, typing was performed on assemblies of insufficient or at least questionable quality. While mostly no ST could be determined in these cases, some were typed, and some even deviated between participants/analyses. This should be preventable by a thorough quality assessment and an upfront exclusion of data that does not fulfill the requirements for “high-quality” and therefore “trustworthy” data.

Regarding the applied schemas, a single 7-gene MLST typing schema per species was clearly preferred while for cgMLST allele typing, a higher variability was evident. For cgMLST focus cluster definitions, the various analysis approaches mainly detected the same or very similar clusters. Again, most differences in focus cluster definition were based on the inclusion or exclusion of samples based on differences in quality assessment. The applied cgMLST tool (mostly ChewieSnake and Ridom SeqSphere+) lead only to minor variations in focus cluster definition although ChewieSnake has a tendency of slightly higher number of AD than analysis with Ridom SeqSphere+ for *L. monocytogenes, S. enterica*, and *E. coli*.

Therefore, when some participants found 11–12 AD to the focus strain of *S. enterica* this led to exclusion of these from focus cluster definition. In these cases, threshold relaxation would detect identical focus cluster to participants using other schemas or tools, as suggested also by previous studies (Coolen et al., 2021).

For *L. monocytogenes, S. enterica*, and *E. coli*, the number of AD was typically higher when larger cgMLST schemas were utilized. Notwithstanding, this had no impact on the focus cluster definition but highlights again the need of adjusting thresholds to schemas and the importance of threshold relaxation.

This trend was particularly pronounced for *C. jejuni*, where the largest differences in cgMLST results were observed. These differences were mainly due to the use of schemas with fewer loci, which yielded lower resolution, and schemas with more loci, which provided higher resolution. During analysis of the participants it turned out that the choice of Camp28 as focus isolate was not optimal as analysis approaches using high resolution schemas were not able to detect it. Obviously, both the organizers and the majority of the participants were not aware of the impact when using high-resolution schemas. In most cases low-resolution schemas were used, probably because they are standard both in Ridom SeqSphere+ and in the documentation of ChewieSnake. The Cody schema contains almost double the number of loci and therefore offers higher resolution. Hence, as a direct consequence, the number of called AD is much larger when applying the Cody schema and not the same thresholds are applicable.

As this inter laboratory study improved awareness, the benefit of higher resolution will be used by larger number of German laboratories in future. The two analysis approaches using Cody scheme here, produced the same clustering results. This reproducibility is in line with a European study (Mixão et al., 2025). Participants using the Cody scheme did not detect any cluster, comprising the here defined focus strain Camp29. However, one of these participants detected another cluster with focus strain Camp04.

In addition to the in-depth detailed analysis of clusters defined by the participants containing the respective focus strains, we also compared all clusters detected, when performing hierarchical clustering on the participants cgMLST-distance matrices. The results were mainly in-line with the focus-cluster analysis, depicting a general large correspondence of partitions where different schemas mostly introduced more variance than different tools. Though the sample size was rather small (1-3 clusters per species) here, the observed trends correlate with studies including larger number of clusters (Mixão et al., 2025).

In conclusion, the different schemas used for the respective species affected the number of called AD larger than different tools used, with the greatest visible effect between cgMLST Cody vs. cgMLST Ridom SeqSphere+ or cgMLST Innuendo schemas for *C. jejuni*.

In concordance with the cgMLST analysis, participants detected very similar focus clusters based on SNP calling. The majority of variance in the number of strains clustering with the focus strain was due to earlier analysis steps, such as quality assessment and MLST. As expected, we observed no differences in the list of clustered samples and their number of SNPs based on the pipelines SnippySnake and WGSBAC (both based on Snippy) and analysis using Snippy directly. Depending on the species, a slight difference was noticed in comparison with the results of CSI Phylogeny. In this study, only one analysis approach (per species) used CSI Phylogeny. However, concordant results between Snippy and CSI Phylogeny were also achieved in a larger study (Mixão et al., 2025). The participants considered different thresholds for inclusion of an isolate in the focus cluster. This was visible for *E. coli* and *C. jejuni/C. coli*, as the clustering was more uniform compared with *L. monocytogenes* and *S. enterica* isolates. Whether participants decided to perform the SNP analysis on trimmed or untrimmed data, no clear difference in clustering results were visible. This might be due to rigorous quality filtering in the Snippy algorithm. As the core genome in the SNP calling depends on the samples included, participants were instructed to perform SNP calling with all strains that have the same 7-gene MLST sequence type as the focus isolate, in an attempt to minimize variability in the results. Performing SNP calling on strains of the same ST has become standard in some European countries (Mixão et al., 2025). The resolution of clustering might be further increased by using ST-specific high-quality reference genomes, which might be assembled combining long- and short-read data. Results were reported that were highly comparable when focus cluster from cgMLST and SNP analyses were compared. The majority of focus clusters found by SNP analysis agreed with those found for cgMLST, while exceptions were mostly based on varying interpretations of quality metrics. To compare all cluster partitions of the various analysis approaches, hierarchical clustering was applied on individual SNP-distance matrices. Very similar clustering results were obtained by the various approaches, with minor outliers. For *C. jejuni*, larger differences were detected due to the use of a recombination filter which is not surprising as recombination events are frequent in *Campylobacter* evolution (Sheppard and Maiden, 2015). The need for filtering these is addressed in Schürch et al. (2018); Uelze et al. (2020b) and genomic epidemiology studies readily apply filters (Ghielmetti et al., 2023; Dziegiel et al., 2024). In summary, even though this study's sample size was relatively small and its analysis approaches largely relied on Snippy, earlier research on SNP analysis produced comparable results (Mixão et al., 2025).

We have previously shown high inter-laboratory reproducibility and accuracy of bacterial genotyping in a proficiency study when participants applied the same library preparation kit, sequencing instrument and bioinformatic software suite with default parameters (Forth et al., 2023). The emphasis of the present inter-laboratory dry-lab investigation was on bioinformatic analysis in order to identify any potential variability caused by different tools or different interpretations of results by the responsible German laboratories. In parallel, an intersectoral assessment of cluster congruence between different bioinformatics pipelines was performed where many European national reference laboratories participated (Mixão et al., 2025). While the European study used larger data sets (sequence data from >2300 isolates) and a larger variety of typing tools (both cgMLST and SNPs), this present study used a larger variety of preprocessing and assembly methods performed by the participants themselves (Mixão et al., 2025). Both studies indicated an overall good cluster concordance when different bioinformatics analysis tools are applied, for both cgMLST and SNP analysis. Moreover, both studies indicate a larger influence on cluster composition from differing cgMLST schemas compared with the use of differing algorithms. Thus, the risk of finding different cluster compositions between two institutes is larger when the institutes used different schemas (but same algorithms) compared to the scenario where same schemas but different algorithms were used. Threshold relaxation increases the chance of detecting identical clusters when comparing different pipelines/tools and schemas. However, care is needed when performing threshold relaxation as false positive strains might be added to outbreak clusters. This present study highlighted another important source of variance, namely the interpretation of results by different persons. In fact, large differences of reported focus clusters were based on differing interpretations on quality metrics. While we expect part of this variance will vanish with users gaining more experience, clear communication is of outmost important. This work provides valuable insights on how to improve the comparability of analysis results between laboratories, as well as collaboration and communication, especially in the context of regular WGS data sharing, analysis and interpretation of clusters in case of prevalent food-borne infections.

## Data Availability

The original contributions presented in the study are included in the article/Supplementary material, further inquiries can be directed to the corresponding authors. Raw sequencing data used in this study is available at: https://zenodo.org/records/14006501.

## Footnotes

^1^ https://github.com/tseemann/shovill

^2^ https://github.com/ablab/spades

^3^ https://github.com/ncbi/SKESA

^4^ https://github.com/raivokolde/pheatmap

^5^ https://www.ridom.de/seqsphere/

^6^ https://gitlab.com/bfr_bioinformatics/bakcharak

^7^ https://github.com/tseemann/mlst

^8^ https://www.cgmlst.org/ncs/schema/Cjejuni104/

^9^ https://www.cgmlst.org/ncs/schema/8896773/

^10^ https://gitlab.com/bfr_bioinformatics/snippySnake

^11^ https://github.com/tseemann/snippy

^12^ https://www.cgmlst.org/ncs/schema/Cjejuni280/

## Supplementary material

Supplementary Figure 1 Selected strains clustering with the focus strain based on cgMLST. **(A)** *S. enterica*, **(B)** *L. monocytogenes*, **(C)** *C. jejuni*, and **(D)** *E. coli*. Individual analyses are named according to the following format: LC_approach_pipeline_assembler_cgMLST-tool_scheme; LC, Laboratory Code (“_A,”… indicates different analysis approaches), Pipelines: Rs, Ridom Seqsphere+; Wg, WGSBAC; Aq, AQUAMIS; Assembler: Sh, Shovill; Sp, Spades; Sk, Skesa; cgMLST tools: Rs, Ridom SeqSphere+; Cs, Chewiesnake; NO, none; Schemas: Eb, Enterobase; Cd, Cody; Rs, Ridom SeqSphere+; In, Innuendo; Mo, Moura; Ru, Ruppitsch; St, STEC.

Supplementary Figure 2 Selected strains clustering with the focus strain based on SNP-typing. **(A)** *S. enterica*, **(B)** *L. monocytogenes*, **(C)** *C. jejuni* and **(D)** *E. coli*. Individual analyses are named according to the following format: LC_approach__SNP-pipeline_SNP-tool; Pipeline: SSN, SnippySnake; Wg, WGSBAC; CSI, CSI Phylogeny; SNP tool: S, Snippy.

Supplementary Figure 3 Adjusted Rand indices as similarity measure comparing clustering based on complete cgMLST-distance matrices provided by the participants. The dendrogram visualizes similarities among analysis approaches. **(A)** *S. enterica*, **(B)** *L. monocytogenes*, **(C)** *C. jejuni* and **(D)** *E. coli*.

Supplementary Figure 4 Adjusted Rand indices as similarity measure comparing clustering based on complete cgSNP distance matrices provided by the participants. The dendrogram visualizes similarities among analysis approaches. **(A)** *S. enterica*, **(B)** *L. monocytogenes*, **(C)** *C. jejuni* and **(D)** *E. coli*.

Supplementary Table 1 Questionnaire regarding methodological details of the interlaboratory study and answers provided by the participants.

Supplementary Table 2 Questions and answers of each participant regarding quality metrics and exclusion of samples.

Supplementary Table 3 Questions and answers of each participant regarding 7-gene MLST analysis and results.

Supplementary Table 4 cgMLST cluster and allelic differences to respective focus strains provided by each participant.

Supplementary Table 5 cgSNP cluster and allelic differences to respective focus strains provided by each participant.
