# Supplementary figures and images for: An inter-laboratory study characterizes the impact of bioinformatic approaches on genome-based cluster detection for foodborne bacterial pathogens
Source: Front Microbiol. 2025 Nov 3;16:1629731. doi: 10.3389/fmicb.2025.1629731 (PMC12621568; doi:10.3389/fmicb.2025.1629731)

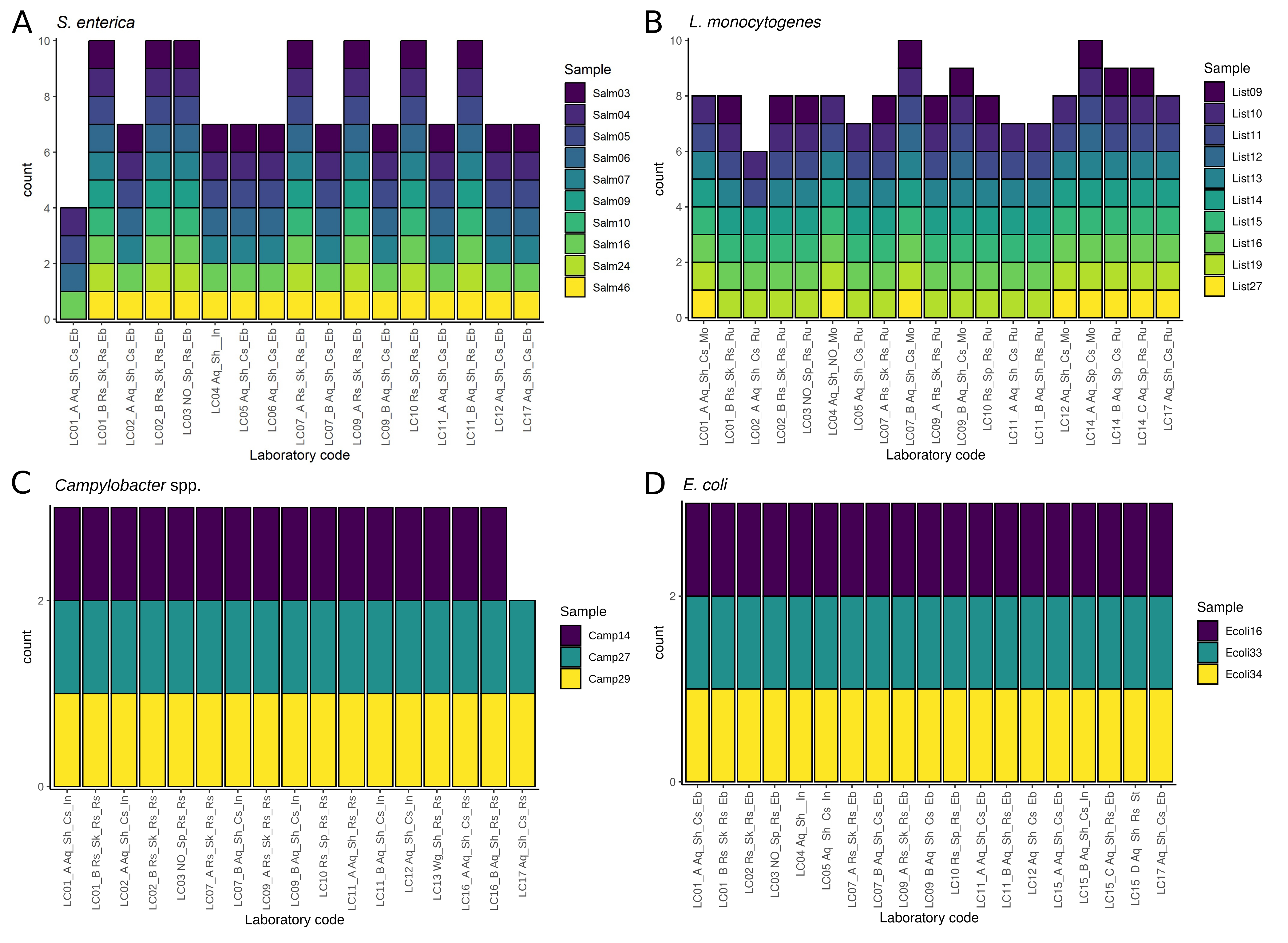

Supplement: Supplementary Figure 1 — Selected strains clustering with the focus strain based on cgMLST. (A) S. enterica, (B) L. monocytogenes, (C) C. jejuni, and (D) E. coli. Individual analyses are named according to the following format: LC_approach_pipeline_assembler_cgMLST-tool_scheme; LC, Laboratory Code (“_A,”… indicates different analysis approaches), Pipelines: Rs, Ridom Seqsphere+; Wg, WGSBAC; Aq, AQUAMIS; Assembler: Sh, Shovill; Sp, Spades; Sk, Skesa; cgMLST tools: Rs, Ridom SeqSphere+; Cs, Chewiesnake; NO, none; Schemas: Eb, Enterobase; Cd, Cody; Rs, Ridom SeqSphere+; In, Innuendo; Mo, Moura; Ru, Ruppitsch; St, STEC. [file Data_Sheet_1.zip › Datasheet 1/Supplementary Figure 1 .png]

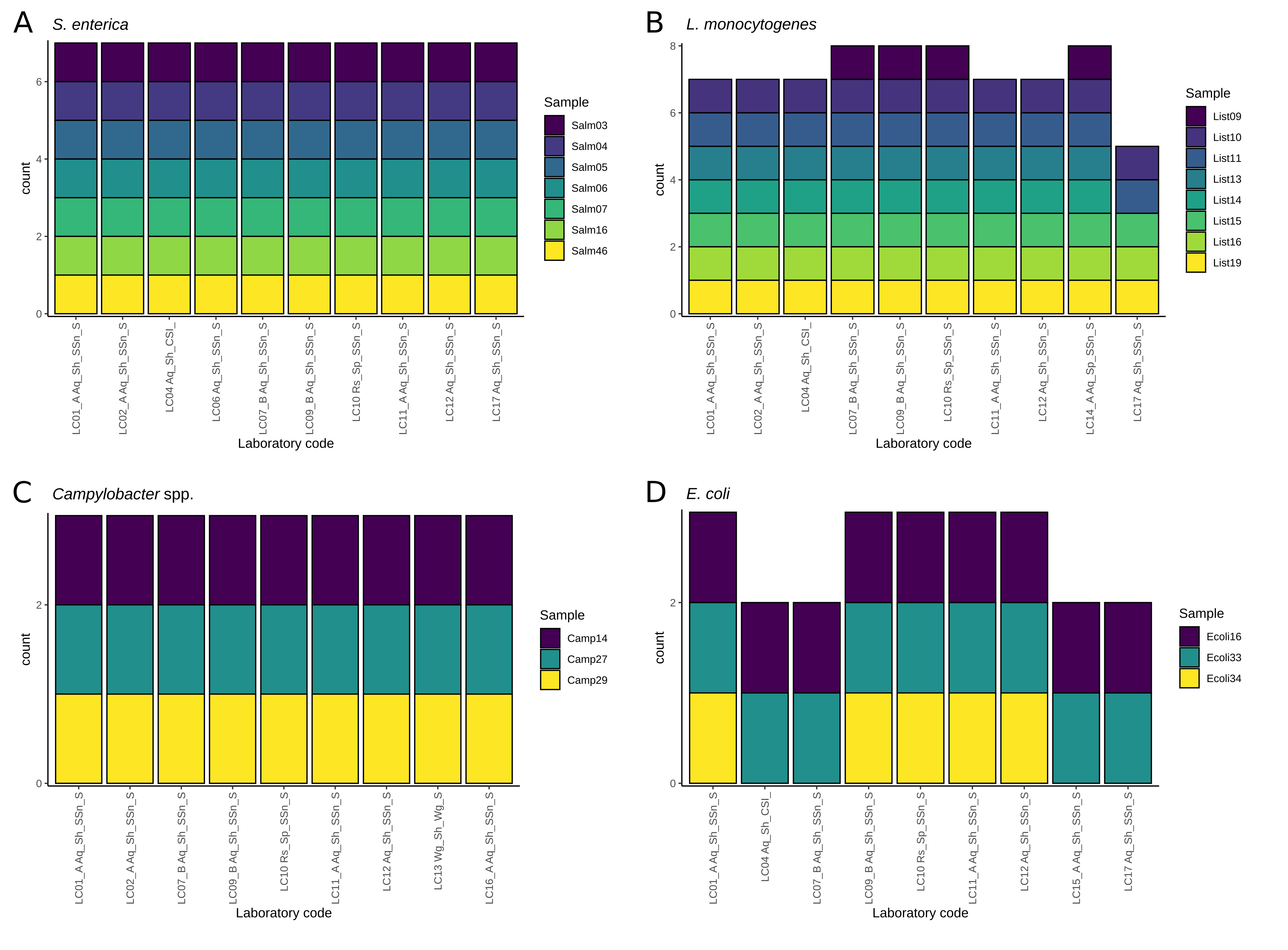

Supplement: Supplementary Figure 1 — Selected strains clustering with the focus strain based on cgMLST. (A) S. enterica, (B) L. monocytogenes, (C) C. jejuni, and (D) E. coli. Individual analyses are named according to the following format: LC_approach_pipeline_assembler_cgMLST-tool_scheme; LC, Laboratory Code (“_A,”… indicates different analysis approaches), Pipelines: Rs, Ridom Seqsphere+; Wg, WGSBAC; Aq, AQUAMIS; Assembler: Sh, Shovill; Sp, Spades; Sk, Skesa; cgMLST tools: Rs, Ridom SeqSphere+; Cs, Chewiesnake; NO, none; Schemas: Eb, Enterobase; Cd, Cody; Rs, Ridom SeqSphere+; In, Innuendo; Mo, Moura; Ru, Ruppitsch; St, STEC. [file Data_Sheet_1.zip › Datasheet 1/Supplementary Figure 2 .png]

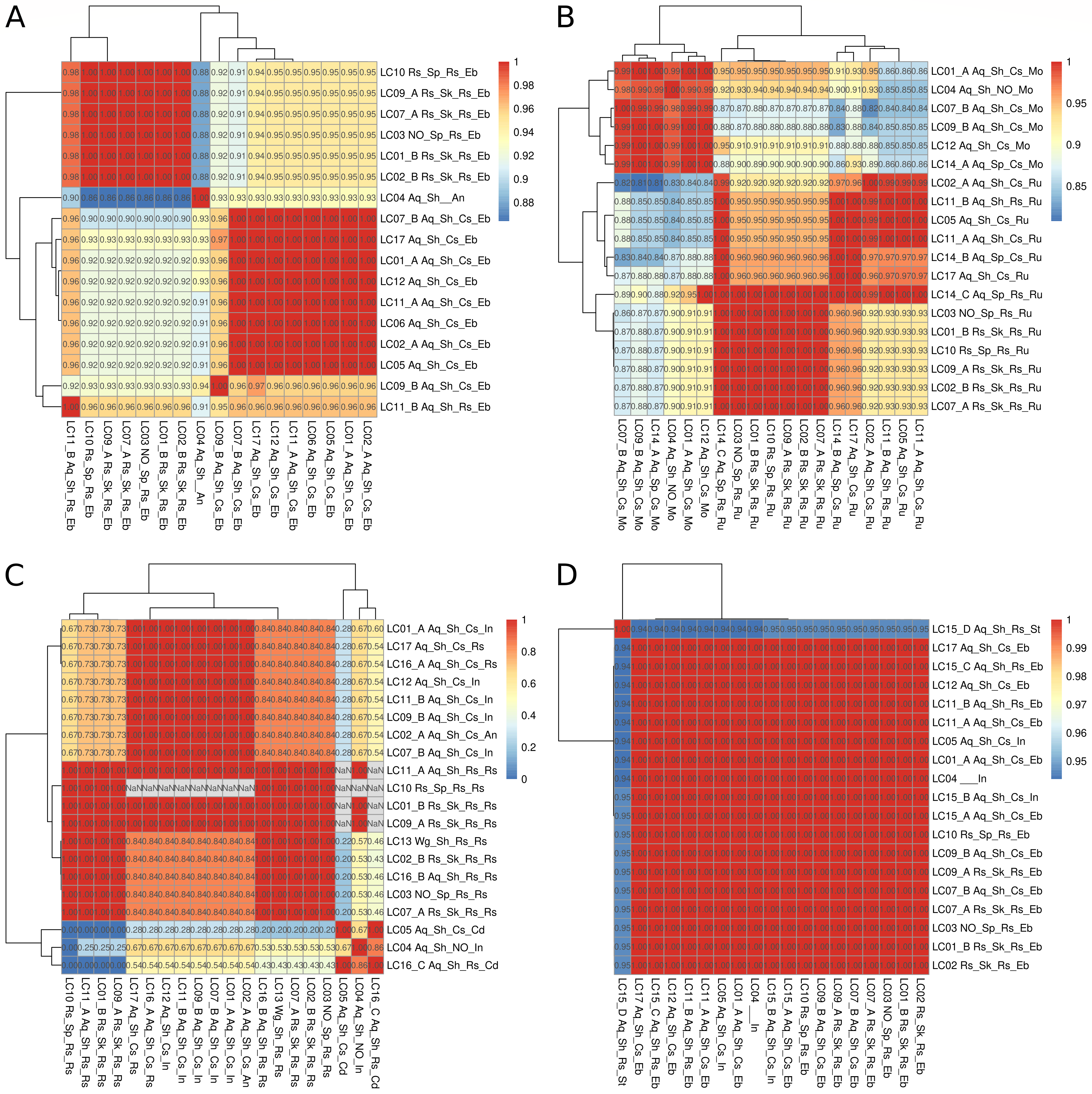

Supplement: Supplementary Figure 1 — Selected strains clustering with the focus strain based on cgMLST. (A) S. enterica, (B) L. monocytogenes, (C) C. jejuni, and (D) E. coli. Individual analyses are named according to the following format: LC_approach_pipeline_assembler_cgMLST-tool_scheme; LC, Laboratory Code (“_A,”… indicates different analysis approaches), Pipelines: Rs, Ridom Seqsphere+; Wg, WGSBAC; Aq, AQUAMIS; Assembler: Sh, Shovill; Sp, Spades; Sk, Skesa; cgMLST tools: Rs, Ridom SeqSphere+; Cs, Chewiesnake; NO, none; Schemas: Eb, Enterobase; Cd, Cody; Rs, Ridom SeqSphere+; In, Innuendo; Mo, Moura; Ru, Ruppitsch; St, STEC. [file Data_Sheet_1.zip › Datasheet 1/Supplementary Figure 3 .png]

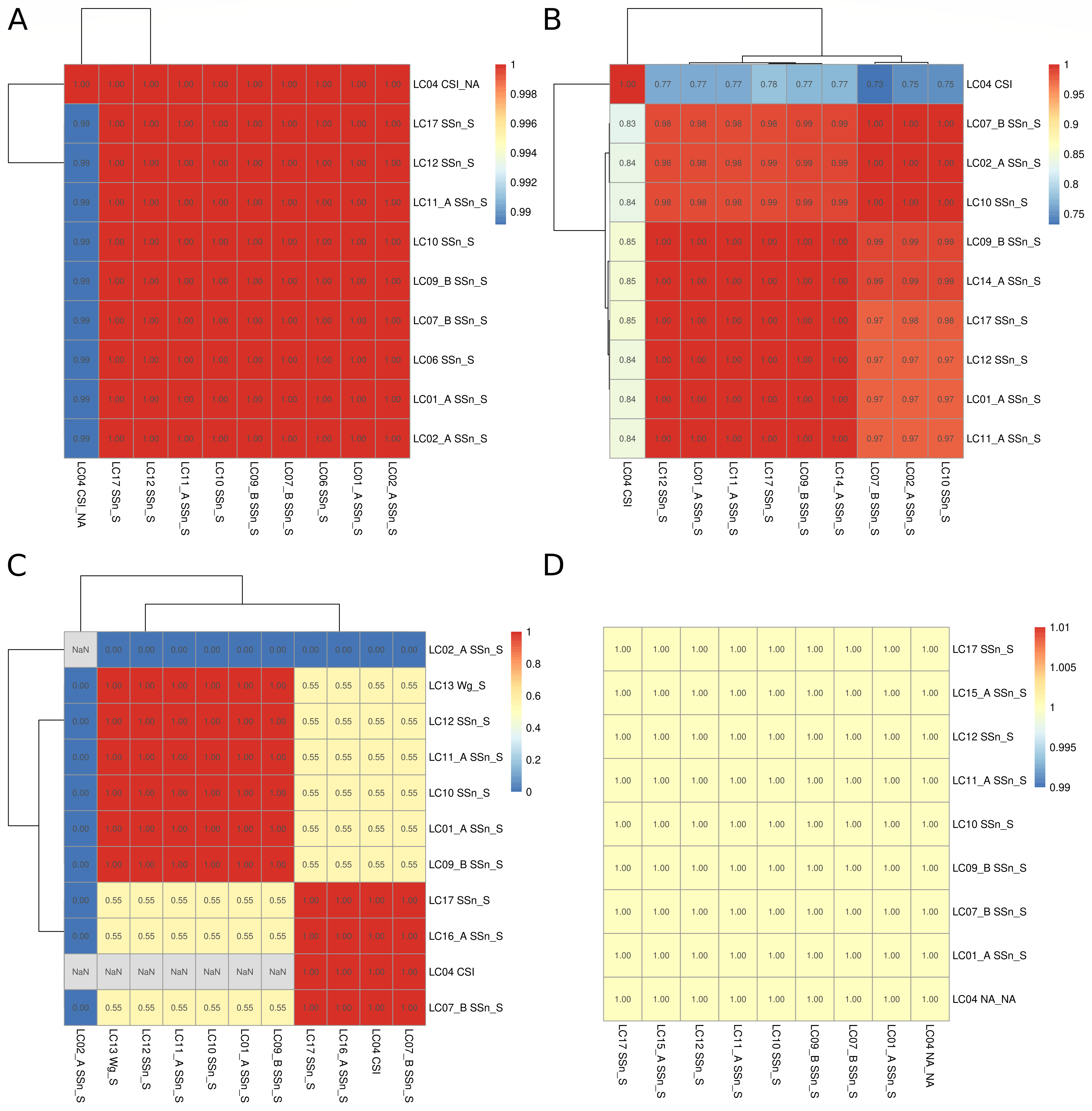

Supplement: Supplementary Figure 1 — Selected strains clustering with the focus strain based on cgMLST. (A) S. enterica, (B) L. monocytogenes, (C) C. jejuni, and (D) E. coli. Individual analyses are named according to the following format: LC_approach_pipeline_assembler_cgMLST-tool_scheme; LC, Laboratory Code (“_A,”… indicates different analysis approaches), Pipelines: Rs, Ridom Seqsphere+; Wg, WGSBAC; Aq, AQUAMIS; Assembler: Sh, Shovill; Sp, Spades; Sk, Skesa; cgMLST tools: Rs, Ridom SeqSphere+; Cs, Chewiesnake; NO, none; Schemas: Eb, Enterobase; Cd, Cody; Rs, Ridom SeqSphere+; In, Innuendo; Mo, Moura; Ru, Ruppitsch; St, STEC. [file Data_Sheet_1.zip › Datasheet 1/Supplementary Figure 4 .png]
